# Drug-Induced Exposure of *Schistosoma mansoni* Antigens SmCD59a and SmKK7

**DOI:** 10.1371/journal.pntd.0003593

**Published:** 2015-03-16

**Authors:** Natalie Reimers, Arne Homann, Beate Höschler, Kristina Langhans, R. Alan Wilson, Christine Pierrot, Jamal Khalife, Christoph G. Grevelding, Iain W. Chalmers, Maria Yazdanbakhsh, Karl F. Hoffmann, Cornelis H. Hokke, Helmut Haas, Gabriele Schramm

**Affiliations:** 1 Division of Cellular Allergology, Priority Area Asthma & Allergy, Research Center Borstel (RCB), Borstel, Germany; 2 Centre for Immunology and Infection, Department of Biology, University of York, York, United Kingdom; 3 Center for Infection and Immunity of Lille, Inserm U1019, CNRS 8204, Institut Pasteur de Lille, Lille, France; 4 Institute for Parasitology, University of Giessen, Giessen, Germany; 5 Institute of Biological, Environmental & Rural Sciences (IBERS), Aberystwyth University, Aberystwyth, United Kingdom; 6 Department of Parasitology, Leiden University Medical Center, Leiden, Netherlands; 7 *helmin*Guard, Suelfeld, Germany; McGill University, CANADA

## Abstract

**Background:**

Schistosomiasis is a serious health problem especially in developing countries and affects more than 243 million people. Only few anthelmintic drugs are available up to now. A major obstacle for drug treatment is the different developmental stages and the varying host compartments during worm development. Anthelmintic drugs have been tested mainly on adult schistosomes or freshly transformed cercariae. Knowledge concerning the larval stages is lacking.

**Methodology/Principal Findings:**

In this study, we used *in vitro*-grown schistosomula (aged between 2 to 14 days) to investigate drug effects of the three anthelmintics praziquantel, artemether, and oxamniquine. Further, we analyzed the antibody accessibility of two exemplary schistosome antigens SmCD59a and SmKK7, before and after drug treatment. Our results demonstrated that praziquantel applied at a concentration of 1 μM inhibited development of all life stages. Application of 10 μM praziquantel led to dramatic morphological changes of all schistosomula. Artemether at 1 and 10 μM had differential effects depending on whether it was applied to 2-day as compared to 7- and 14-day schistosomula. While 2-day schistosomula were not killed but inhibited from further development, severe morphological damage was seen in 7- and 14-day schistosomula. Oxamniquine (1 and 10 μM) led to severe morphological impairment in all life stages. Analyzing the accessibility of the antigens SmCD59a and SmKK7 before drug treatment showed no antibody binding on living intact schistosomula. However, when schistosomula were treated with anthelmintics, both antigens became exposed on the larvae. Oxamniquine turned out to be most effective in promoting antibody binding to all schistosomula stages.

**Conclusion:**

This study has revealed marked differences in anthelmintic drug effects against larvae. Drug treatment increases surface antigen presentation and renders larvae accessible to antibody attack.

## Author Summary

Schistosomiasis is one of the major parasitic diseases in developing countries and still causes 200,000 deaths per year. Mass drug administration programs with praziquantel, the drug of choice against schistosomiasis, are currently undertaken in Sub-Saharan Africa. Praziquantel, although efficient against adult worms, fails to cure early infection. The complex developmental stages of schistosomes and migration through varying host compartments with different local drug concentration are a challenge for drug treatment. After infecting their mammalian host, schistosomula traverse through skin and the vasculature of lung, liver and intestines. During their migration, they develop from larvae to paired adults in approximately 4 to 5 weeks. So far, drug effects have been analyzed on adult worms or freshly transformed schistosomula only. Information about the effects on the larval stages is lacking. We were able to transfer the larval development of the first three weeks into the culture dish. This tool can be used for the analysis of drug effects against schistosomula and for investigation of the accessibility, expression and localization of antigens. Rendering the parasite’s larvae vulnerable to the host’s immune system by increasing antigen presentation is an important aspect of drug activity. We demonstrate on *in vitro*-cultured *Schistosoma mansoni* larvae, that SmCD59a and SmKK7, as examples for hidden antigens, become accessible to antibodies following drug treatment.

## Introduction

Schistosomiasis is one of the major parasitic diseases affecting people in tropical and subtropical countries. In endemic areas, recurring reinfection may lead to lingering illness and life-threatening complications. In children, serious developmental disorders like growth retardation or complications like ascites can be the result of a schistosome infection [1]. According to the WHO, 243 million people in 78 countries required treatment in 2011 [2]. There are only a few drugs available against schistosomiasis. Praziquantel (PZQ), which is efficient against adult worms, but does not kill larvae or prevent reinfection [3], is the drug of choice. Mass drug administration programs with PZQ are being undertaken in Sub-Saharan Africa [2]. Consequently, the possible development of PZQ-resistant parasites is one of the current concerns for schistosomiasis treatment and prevention [4]. Drug resistance has already been reported in parasite populations derived from infected individuals treated with oxamniquine (OXA). The drug had been used extensively in endemic areas; during a national disease control program in Brazil, over 12 million doses were applied [5]. But as resistant schistosomes prevailed, it has not been used since 2010 [6]. Due to restricted numbers of chemotherapeutic options for treating schistosomiasis, novel drugs or new approaches are urgently needed. Searches for next-generation anti-schistosomals have led to investigating the properties of antimalarial drugs, as haematophagus *Plasmodium* and *Schistosoma* parasites both need to detoxify the damaging actions of haem [7]. For example, artemisinin derivatives like artemether (ART), which are used against malaria, have been shown to effectively treat schistosomiasis [8]. However, ART is not as effective as is PZQ when it is applied as monotherapy [9]. Consequently, the WHO has not recommended artemisinin derivatives for schistosomiasis treatment [10].

A major obstacle for effective drug treatment and drug development is the schistosome life cycle and the altering susceptibility to drugs depending on the developmental stage [3]. After infecting their mammalian host, schistosomes traverse through skin, vasculature of lung, liver and intestines. During their migration, they develop from larvae to paired adults in approximately 4 to 5 weeks [1]. Each stage is known to vary its antigenic profile [11]. So far, experimental analysis of drug effects has been done with adult worms or freshly transformed schistosomula. However, there is a lack of information on drug effects on the more mature larval stages. Analysis of the developing larvae *in vivo* is difficult, consuming both time and animals. Thus, we used *in vitro*-cultured schistosomula. These were grown under conditions chosen according to the protocol of Basch [12]. This approach allows continuous monitoring of maturation and viability of the larvae and is, therefore, a suitable tool for detailed analysis of drug effects and antibody binding on developing schistosome larvae. Schistosomes have developed strategies to remain hidden from the damaging activities of the mammalian immune system. For example, as the antigenic profile changes throughout the life cycle, surface antigens can be masked from host recognition [11]. However, effective schistosomiasis treatment depends on the immune response of the host to the parasitic infection: The immune status of the host has been shown to affect the outcome of treatment [13,14]. Drugs like PZQ possibly trigger an immune response by making the schistosomula susceptible to antibody attack through increased presentation of surface antigens [3].

In this study, we examined the surface antigen SmCD59a and SmKK7, an antigen localized to the nervous system [15]. SmCD59a is a GPI-anchored schistosomal tegument protein, which was found to be expressed in *S*. *mansoni* in various life stages from cercariae up to adults [16]. SmCD59a is one of six homologues of human CD59, an inhibitor of the complement system membrane attack complex [17]. Despite sequence identity to the human CD59 of between 20 and 30% and the presence of the characteristic three-fingered protein domain (TFFD), detailed characterization of two of the CD59-like members, SmCD59a and SmCD59b, revealed no complement inhibition activity, and their function in schistosomes is still unknown [17–19]. SmKK7 is a potential immunomodulator with homology to the potassium channel blockers in scorpion venom [20]. Cercariae secrete SmKK7 [20] and high SmKK7 expression was seen in the sensory nerve endings on the anterior surface of the cercariae and adult worms [15]. Inside the schistosomes, SmKK7 is distributed in the peripheral nervous system [15].

Rendering larvae accessible to antibody attack is an important property of drug activity. The aim of this study thus was to investigate the accessibility of the antigens SmCD59a and SmKK7 after pharmacological treatment of *in vitro*-cultured schistosomula with PZQ, ART, and OXA. Accessibility, expression and localization of SmCD59a and SmKK7 were studied at different stages of development. On living intact schistosomula, SmCD59 and SmKK7 are not bound by antibodies against these proteins. In contrast, antibodies bind to the surface of formalin-fixed and permeabilized schistosomula and also to the surface of drug-exposed schistosomula.

## Materials and Methods

### Ethics statement

All animal experiments have been performed in accordance with the European Convention for the Protection of Vertebrate Animals used for experimental and other scientific purposes (ETS No 123; revised Appendix A) and have been approved by the Regional Council (Regierungspraesidium) Giessen (V54–19 c 20/15 c GI 18/10) or the Procedure Comitéd’Ethique en Experimentation Animal Nord Pas de Calais; CEEA 142010. Procedures in York involving animals were carried out in accordance with the UK Animals (Scientific Procedures) Act 1986 and authorized on personal and project licences issued by the UK Home Office. The vaccination protocol was approved by the Biology Department Ethical Review Committee.

### *In vitro* culture of *S*. *mansoni*

The *S*. *mansoni* life cycle has been established in our laboratory according to previously published protocols [21]. As intermediate host, *Biomphalaria glabrata* snails (Puerto Rican strain) were infected with miracidia. The latter hatched from eggs derived from livers of Syrian hamsters (*Mesocricetus auratus) infected with a Liberian strain of S*. *mansoni*. Cercariae were obtained from *B*. *glabrata* by light exposure 6 weeks after infection. Transformation was performed mechanically *via* repeated passages through an emulsifying needle. The transformed schistosomula were cultured as described previously by Basch et al. [12], yet with slight modifications to the method: Schistosomula were cultured in Iscove’s modified Dulbecco’s medium (PAA Laboratories GmbH) containing 5 μg/ml insulin (Gibco), 50 μg/ml transferrin (Sigma Aldrich), 100 U/ml penicillin (PAA), and 100 μg/ml streptomycin (PAA) with 10% fetal calf serum, FCS (PAA). After 24 h, human peripheral blood mononuclear cells (PBMC) from healthy donors were added at a concentration of 8 × 10^2^ cells per μl. After 48 h, human serum (final concentration 20%) and whole blood (0.3%) were added to the culture system. Schistosomula were cultured at 37°C and 6% CO_2_ for up to 21 days.

### Sera from infected and immunized rats

To obtain rat infection sera (RIS), male 8-week-old inbred Fischer F344 or BN rats (Charles River) were percutaneously exposed to 2 × 10^3^cercariae as previously described (primary infection) [22] and re-exposed to 2 × 10^3^cercariae after 63 days (secondary infection). Sera were recovered 3 weeks after the secondary infection, pooled and used as positive control (RIS).

Monospecific rat anti-SmCD59a antiserum was raised against SmCD59a that was recombinantly expressed in *Pichia pastoris*: RNA was extracted from adult worms using Trizol reagent according to the manufacturer's instructions. The first cDNA strand was made using a Superscript III kit (Invitrogen) primed with poly-T. The full length SmCD59a, minus the signal peptide, was cloned into a pPICz alpha expression vector (Invitrogen) using specific primers. *Pichia* clones were isolated and bulk expression was performed according to the manufacturer's instructions (Invitrogen). Protein was purified from the fermentation supernatant by affinity chromatography on a nickel column and dialyzed into PBS. Two Wistar female rats were immunized with a 100 μg purified recombinant protein emulsified in 100 μl Titermax Gold adjuvant (Sigma-Aldrich), administered subcutaneously on the back of the neck, with two subsequent boosts at 3 week intervals before a terminal bleed at 8 weeks.

Monospecific rat anti-SmKK7 antiserum was raised against SmKK7 recombinantly expressed in *E*. *coli*. RNA was extracted from adult worms using Trizol reagent according to the manufacturer's instructions. The first cDNA strand was made using a Superscript III kit (Invitrogen) primed with random hexamers. The full length SmKK7, minus the signal peptide, was cloned into a modified pET30a vector (Novagen) using specific primers. The pET30a/SmKK7 plasmid was transformed into chemically competent E. coli BL21 star (DE3) cells (Invitrogen) and expression of rSmKK7 followed the protocols listed in the BL21 star (DE3) manual. Bacteria were pelleted and lysed 3hafter isopropyl β-D-1-thiogalactopyranoside (IPTG) induction (0.5 mM final concentration), and the resultant soluble fraction was then used to purify the recombinant protein. rSmKK7 was purified from the soluble fraction using Ni-NTA agarose beads (Qiagen) according to the manufacturer’s instructions and dialyzed into PBS.

### Western blot analysis of *S*. *mansoni* extracts

To investigate the expression of SmCD59a and SmKK7 at different time points after transformation, schistosomula were cultured *in vitro* for 2, 7, or 14 days as described above. Schistosomes were harvested, lysed by adding NP-40 lysis buffer (1% Nonidet P-40, 150 mM NaCl, 50 mM Tris-HCl, pH 7.4) and homogenized mechanically. Equal amounts of total protein (1 μg) were separated by 12% SDS-PAGE under non-reducing conditions and blotted onto a nitrocellulose membrane. Free binding sites on the membrane were blocked with 0.1 M Tris-HCl, pH 7.4 containing 0.05% Tween (blocking buffer). The membrane was incubated overnight with rat antisera directed against SmCD59a and SmKK7, respectively, diluted 1:500, followed by a 2-h-incubation with an alkaline-phosphatase-labeled goat anti-rat IgG (Dianova) in a 1:10,000 dilution. Antibody dilutions and washing steps were performed in blocking buffer. Antibody binding was visualized by a substrate/chromogen mixture of 0.033% (w/v) nitro blue tetrazolium and 0.017% (w/v) 5-bromo-4-chloro-3-indolyl-phosphate (Serva) in TRIS-buffered saline, pH 9.5 [23]. To demonstrate equal loading and blotting, proteins were stained with India Ink (Pelikan) in a 1:1,000 dilution for 60 min, following two washes with blocking buffer.

### Immunofluorescence analysis

*In vitro*-cultured living schistosomes were washed three times with phosphate buffered saline, PBS (PAA) and incubated with anti-SmCD59a or anti-SmKK7 antiserum, normal rat serum (NRS, negative control) or rat infection serum (RIS, positive control) for 1 h. All sera were diluted in a 1:50 ratio. After a washing step, schistosomes were incubated with Alexa594-labeled anti-rat IgG (F(ab)_2_ fragment, Invitrogen) in a 1:500 dilution for 30 min, washed twice and transferred to a chamber slide (Ibidi) for fluorescence microscopic analysis. Where indicated, schistosomes were fixed in 4% formaldehyde in PBS for 15 min at RT, followed by three washes with PBS. Samples were permeabilized with PBS containing 0.1% Triton X-100 for 15 min. Non-specific binding was blocked by incubation for 30 min in PBS containing 1% BSA. Fluorescence microscopy was performed at Olympus IX and Leica SP5 (confocal).

### *In vitro* drug assays

For testing the effects of anthelmintic drugs, *in vitro* schistosomes were cultured as described above. The respective drugs were added directly to the culture system after 2, 7 or 14 days. The final concentrations of the drugs were 0.01, 0.1, 1 and 10 μM. As the drugs were dissolved in DMSO, schistosomula were cultured in medium containing 0.1% DMSO for control (corresponding to the final DMSO content when the highest drug concentration was added to the culture). Schistosomes were incubated for 2 days with PZQ or ART (Selleckchem.com) or for 6 days with OXA prior to analysis. PZQ and OXA were kindly provided by Prof. Dr. Donato Cioli. Changes of the morphology and motility of the schistosomula were monitored by microscopy.

## Results

### *In vitro* development of *S*. *mansoni*

**Fig 1:**
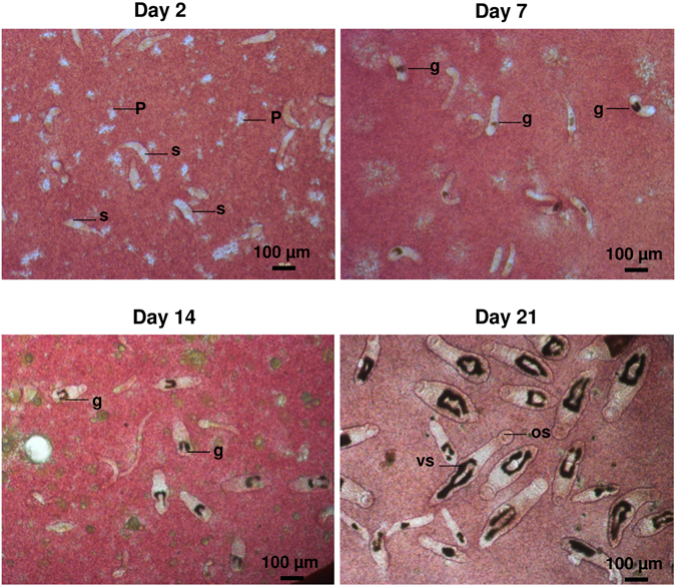
Development of *in vitro*-cultured schistosomula. ***S*. *mansoni* larvae were cultured *in vitro* in IMDM cell culture medium supplied with human PBMC, serum and whole blood.** Images were taken at day 2, 7, 14 and 21 after transformation. By day 7 the gut (filled with black hemozoin) had developed; by day 14 the typical divided gut became clearly visible, and by day 21 oral and ventral suckers appeared. PMBC (P), s (schistosomula), g (gut), os (oral sucker), vs (ventral sucker).

To monitor schistosomal drug effects and antibody binding to developing schistosome larvae, we established the *in vitro* culture of *S*. *mansoni* according to Basch [12]. Parasite development was assessed by parameters such as shape, growth rate, gut development, and motility including blood-feeding behavior. Fig. 1 shows the development of the schistosomula at different time points over a 3-week period after transformation. By day 7, schistosomula showed gut development as indicated by the intestinal accumulation of hemozoin. By day 14, the bifurcated proximal gut, typical for this life stage, was clearly visible. Over the next 7 days, the worms doubled their size from approximately 250 μm to 500 μm. By day 21, half of the schistosomula showed gut fusion near the ventral sucker. Thus, the development of our *in vitro*-cultured larvae matches reports for *ex vivo* schistosomula of the same age [24].

### Anthelmintic drugs PZQ, ART and OXA induce distinct damage and abnormal phenotypes of *in vitro*-cultured schistosomula

**Fig 2:**
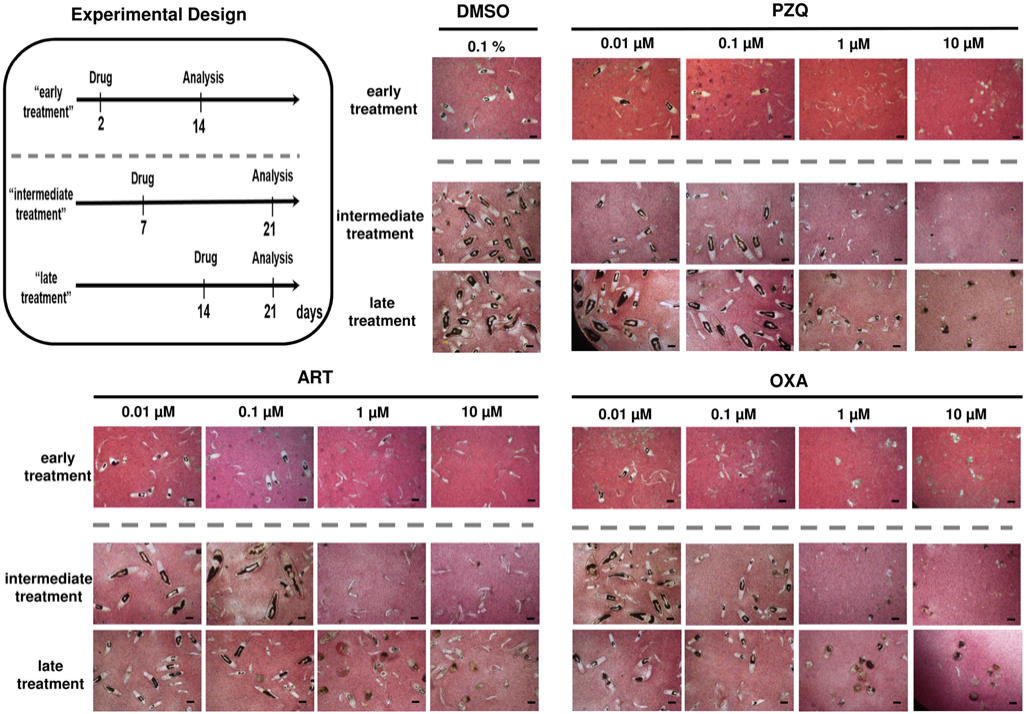
Schistosomicidal effects of PZQ, ART, and OXA on 2-, 7- and 14-day schistosomula. Schematic presentation of the experimental design and morphology of *in vitro*-cultured schistosomula treated with PZQ, ART, and OXA at 0.01, 0.1, 1 and 10 μM. Microscope images were taken at day 14 (early treatment) or day 21 (intermediate and late treatment) as indicated. 0.1% DMSO in medium (solvent of the drugs; used as negative control) had no effect on schistosomula development (representatives out of triplicate wells are shown, scale bars = 100 μm).

**Fig 3:**
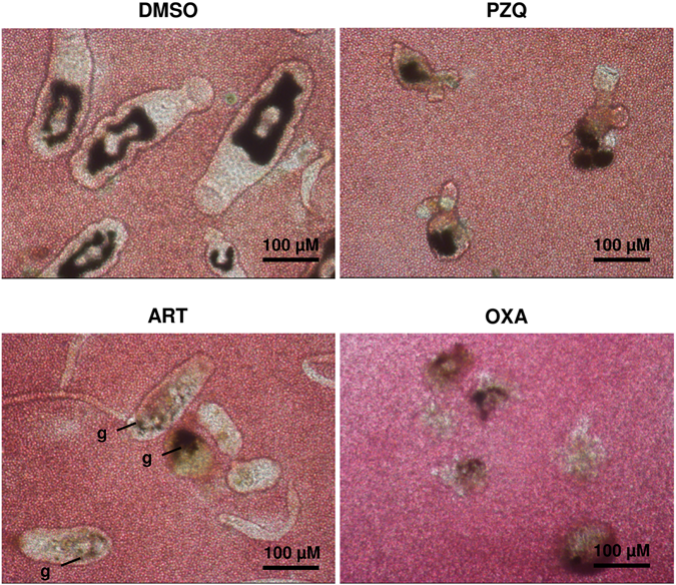
Schistosomicidal effects of PZQ, ART and OXA on 14-day-schistosomula. Magnification of the *in vitro*-cultured schistosomula treated with PZQ, ART, and OXA at 10 μM. Drugs were added at day 14 and schistosomula were analyzed at day 21 (late treatment). 0.1% DMSO in medium (solvent of the drugs; used as negative control) had no effect on schistosomula development (representatives out of triplicate wells are shown; see also Fig. 2 and S2–S5 Videos).

Schistosomicidal drug effects against larvae were assessed by application of the anthelmintic drugs PZQ, ART and OXA, which were added individually to 2-, 7- and 14-day *in vitro-*cultured schistosomula (referred to as “early, intermediate and late treatment” in Fig. 2, Experimental Design). In a first step, their effects on morphological integrity, growth and gut development were analyzed at day 14 (“early treatment”) or 21 (“intermediate and late treatment”) following transformation. Since the drugs were dissolved in DMSO, possible harmful effects of this solvent on worm development were determined as negative control. No adverse effects were seen at the final concentration of 0.1%, the same as in drug-treated wells (S1 Video). PZQ applied at concentrations of 0.01 or 0.1 μM caused no effects. At a concentration of 1 μM, PZQ enhanced the proportion of schistosomula without visible guts compared to the control. Application of 10 μM PZQ led to dramatic morphological changes of all schistosomula (see also Fig. 2. and Fig. 3). Motility was significantly reduced, although it was still detectable (S2 Video). The effect of ART at high concentrations (1 and 10 μM) differed depending on its application to 2-day or to 7- and 14-day schistosomula. Early-treated schistosomula were only subject to inhibition of their further development but remained alive. However, severe morphological damage was seen in intermediately and late treated schistosomula, which have already developed a gut and feed on erythrocytes (Fig. 3g and S3 Video). OXA delayed schistosomal development already at 0.1 μM. When applied at high concentrations (1 and 10 μM), OXA led to severe morphological impairment at all life stages with almost complete disintegration (Fig. 2 and Fig. 3) and loss of motility (S4 Video). Taken together, the *in vitro* approach allows for evaluation of the severity of anthelmintic drug damage on developing schistosomes and for discrimination of the effects by the developmental stage of the schistosomes. Among the three drugs under study, OXA had the most pronounced effect. We further examined whether treatment of the schistosomula with the three drugs under study leads to exposure of surface antigens. As exemplary antigens we chose SmCD59a (Smp_019350) and SmKK7 (Smp_194830) [15,17,18,20].

### Differential expression and distribution of the antigens SmCD59a and SmKK7 of *in vitro*-cultured schistosomula during development

**Fig 4:**
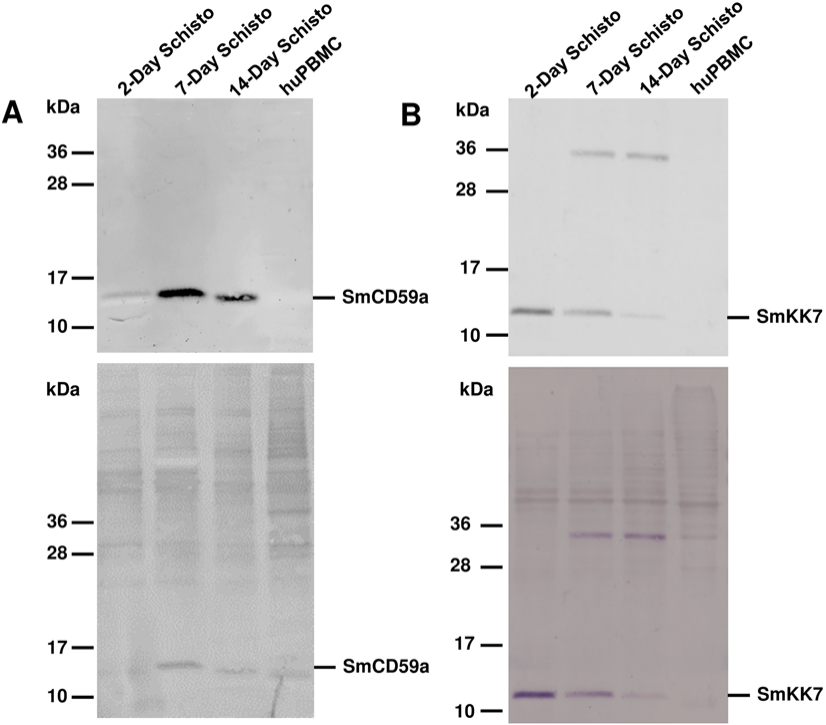
Expression of SmCD59a and SmKK7 in extracts of 2-, 7- and 14-day schistosomula. Western blots of extracts of 2-, 7- and 14-day schistosomula were incubated with rat anti-SmCD59a antiserum (upper panel A) and rat anti-SmKK7 antiserum (upper panel B), binding of IgG antibodies were detected with labeled anti-rat IgG. No cross-reactivity was observed with extract of human PBMC (huPBMC). Same blots subsequently stained with India Ink for protein detection (lower panel A+B).

**Fig 5:**
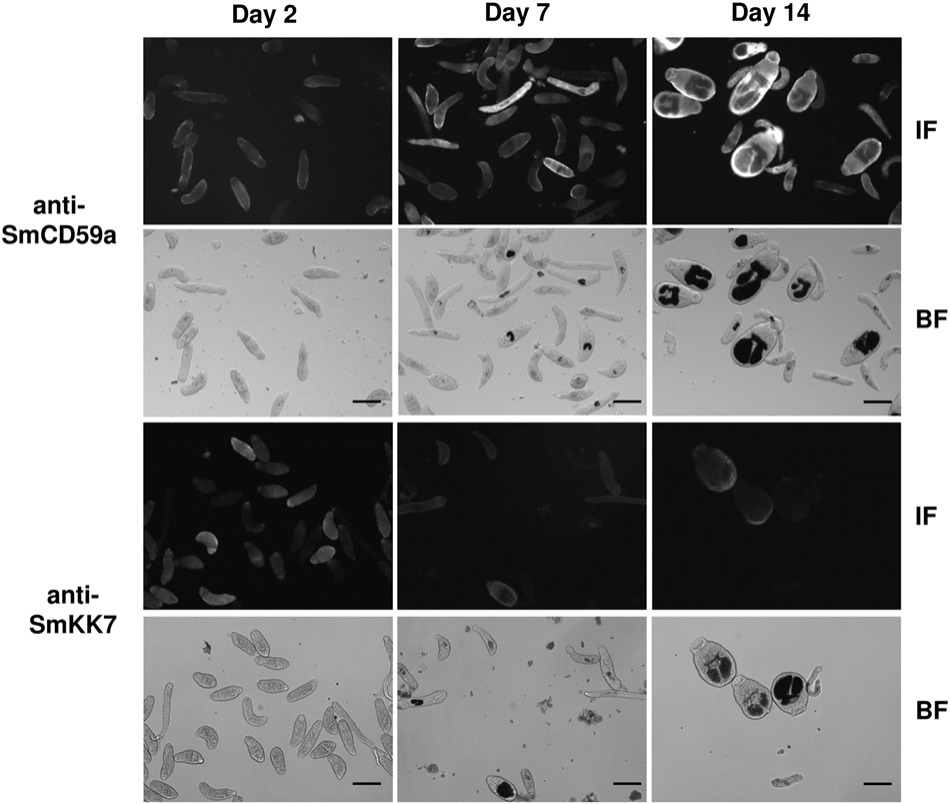
Immunohistochemical analysis of SmCD59a and SmKK7 expression in schistosomula. Formalin-fixed and permeabilized schistosomula were analyzed by immunofluorescence microscopy at day 2, 7 and 14 following transformation. Schistosomula were incubated with rat anti-SmCD59a antiserum and rat anti-SmKK7 antiserum, binding of IgG antibodies were detected with fluorophore-labeled anti-rat IgG. The IF marked panels show the SmCD59a and SmKK7 fluorescence signal. Corresponding lower panels show the bright field image for control of the larval development (BF). Scale bars = 100 μm.

**Fig 6:**
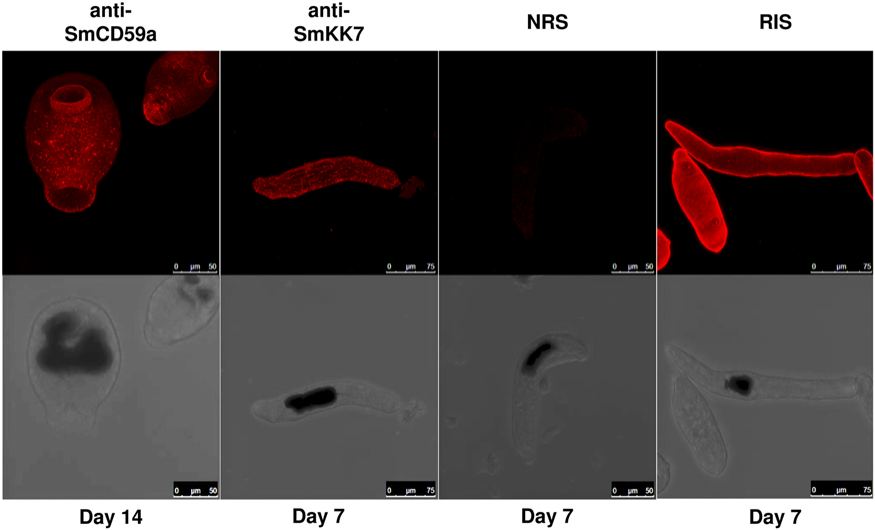
3D projection of schistosomula reveals differential localization of SmCD59a and SmKK7. Formalin-fixed and permeabilized schistosomula were analyzed at day 14 for SmCD59a localization and at day 7 for SmKK7 localization. Schistosomula were incubated with rat anti-SmCD59a antiserum, rat anti-SmKK7 antiserum, a control sample from uninfected rats (NRS) and a control sample from infected rats (RIS). Binding of IgG antibodies were detected with fluorophore-labeled anti-rat IgG. Immunofluorescence was visualized by projection overlay of a confocal z-stack. Corresponding panels show the bright field image for control of larval development.

For a distinct evaluation of the drug-induced exposure of SmCD59a and SmKK7, we first analyzed expression and localization of the two antigens during development. To characterize the expression of SmCD59a and SmKK7, Western blot analysis of extracts from 2-, 7- and 14-day schistosomula was performed using rat antiserum raised against SmCD59a (recombinantly expressed in yeast *P*. *pastoris*) or SmKK7 (recombinantly expressed in *E*. *coli*). Both antisera bind specifically to distinct bands corresponding to the putative size of the respective natural antigen (SmCD59a: 16 kD, SmKK7: 12 kD). The additional band at 36 kD is presumably an artefact due to expression in *E*. *coli* (Fig. 4B). Noteworthy, expression of the two investigated antigens varies over time during schistosome development. SmCD59a is only faintly seen in extracts of 2-day schistosomula and is strongly present in extracts of 7- and 14-day schistosomula. Inversely, the expression of SmKK7 is strongest in 2-day schistosomula and decreases with the larvae growing older. Both antisera did not cross-react with human CD59 or other proteins in the extracts from human PBMC, which were used as a negative control. For immunohistological detection of the two antigens, formalin-fixed and permeabilized schistosomula were incubated with antisera against SmCD59a and SmKK7. After staining with a labelled secondary antibody, 2-, 7-, and 14-day *in vitro*-cultured schistosomula were analyzed by fluorescence microscopy. In line with the results of the Western blots, SmCD59a fluorescence intensity was stronger on 7-day and 14-day schistosomula than on 2-day schistosomula, while SmKK7 staining was most intense on 2-day schistosomula (Fig. 5). Analysis of the antigen localization by confocal microscopy and a projection of a z-stack revealed that in permeabilized schistosomula SmCD59a accumulated near the tegumental surface (Fig. 6, S5 Video). SmCD59a is distributed more diffusely on the schistosomula surface, while SmKK7 appears in condensed spots (Fig. 6, S6 Video). The staining pattern was compatible with the observation that SmKK7 is arranged as part of an internal network [15]. The schistosomula were not stained with the negative control NRS (S7 Video). In contrast, staining with RIS as a positive control resulted in a bright fluorescent signal at the schistosomal surface (S8 Video).

### The antigens SmCD59a and SmKK7 are not accessible on living intact schistosomula, but become exposed after drug treatment

**Fig 7:**
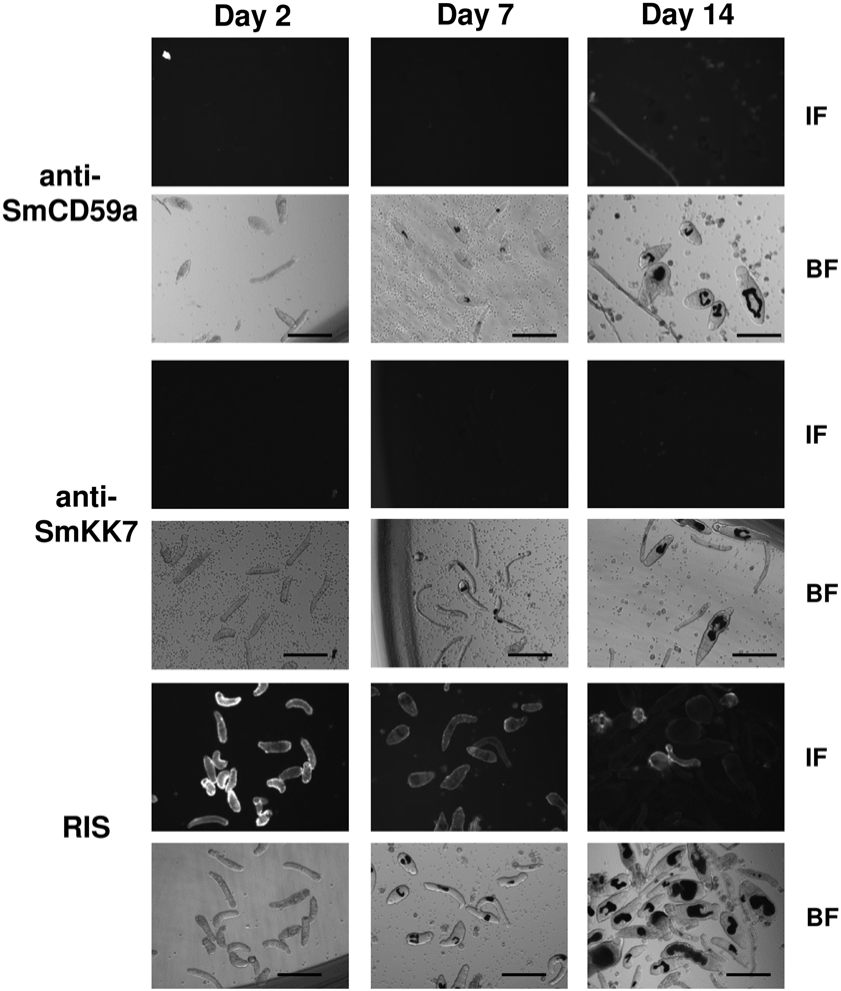
SmCD59a and SmKK7 on living, intact schistosomula are not accessible by anti-SmCD59a and anti-SmKK7 antisera. Binding of rat anti-SmCD59a antiserum (upper panel), rat anti-SmKK7 antiserum (median panel) and serum from infected rat (RIS; lower panel) to schistosomula at day 2, day 7 and day 14 after transformation. Antibody binding was detected with a fluorophore-labeled anti-rat IgG (IF). Corresponding lower images show the bright field images for control larval development (BF). Anti-SmCD59a and anti-SmKK7 antisera do not recognize the antigens on the surface of the live schistosomula, while the RIS shows strong binding, strongest on day 2. Scale bars = 100 μm.

For detecting SmCD59a and SmKK7 on living larvae immunofluorescence microscopy was performed on 2, 7-, and 14-day schistosomula, using the respective specific rat antisera and a fluorophore-coupled secondary antibody. As outlined above, we could detect both antigens in blotted extracts of schistosomula (Fig. 4) and on formalin-fixed and permeabilized larvae (Fig. 6). No antibody binding could be detected on the surface of living intact 2-, 7- and 14-day *in vitro*-cultured schistosomula (Fig. 7). In contrast, RIS used as a positive control gave a bright fluorescent signal at the schistosomal surface, strongest on 2-day schistosomula (Fig. 7). Notably, single mechanically damaged or otherwise impaired schistosomula in the culture are also fluorescent—the former at the lesion sites—suggesting exposure of otherwise hidden antigens (S1 Fig.).

**Fig 8:**
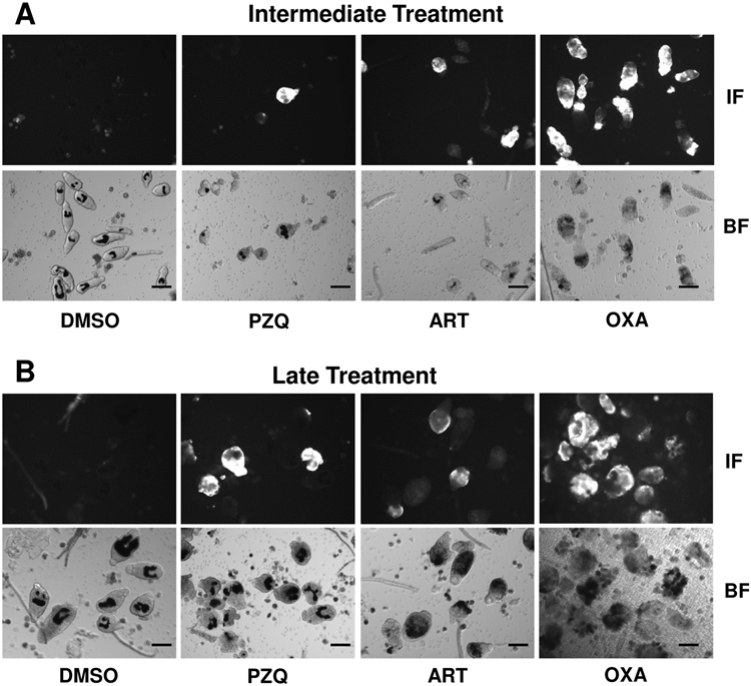
Differential unmasking of the surface antigen SmCD59a by treatment with PZQ, ART and OXA. Drugs were added either at day 7 or day 14 and analyzed 2 days after administration of PZQ and ART and 6 days after OXA application. For the analysis of antigen accessibility, schistosomula were incubated with anti-SmCD59a antiserum. Antibody binding was detected with a fluorophore-labeled anti-rat IgG (IF). Corresponding lower images show the bright field images (BF). Scale bars = 100 μm.

**Fig 9:**
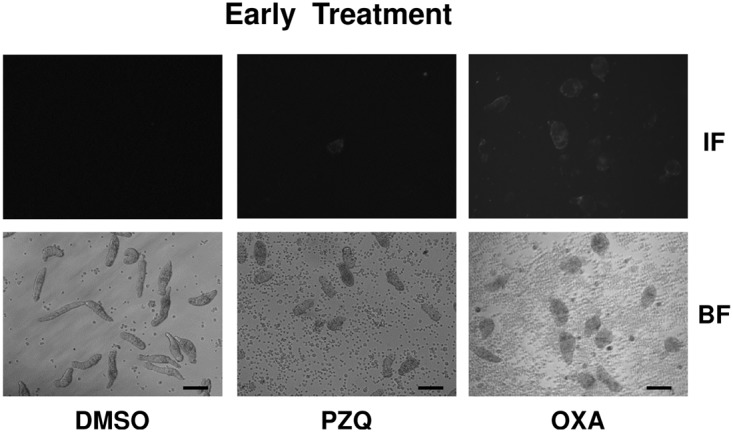
Differential unmasking of the antigen SmKK7 by treatment with PZQ and OXA. Drugs were added at day 2 and analyzed 2 days after administration of PZQ and 6 days after OXA application. For the analysis of antigen accessibility, schistosomula were incubated with anti-SmKK7 antiserum. Antibody binding was detected with a fluorophore-labeled anti-rat IgG (IF). Corresponding lower images show the bright field images (BF). Scale bars = 100 μm.

**Fig 10:**
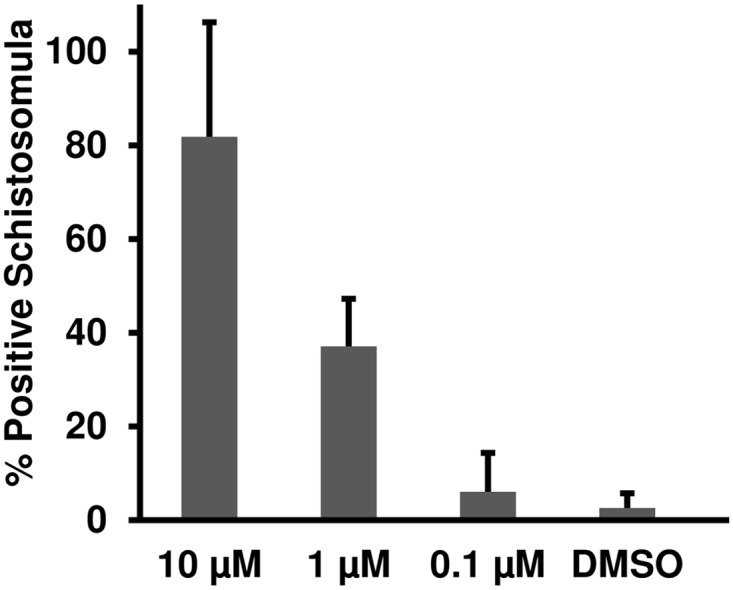
Percentage of unmasked schistosomula after treatment with different OXA doses. 14-day schistosomula were treated with 10, 1 or 0.1 μM OXA. 6 days after drug application schistosomula were incubated with anti-SmCD59a antiserum. Antibody binding was detected with a fluorophore-labeled anti-rat IgG and positive larvae were counted under an inverted fluorescence microscope. Data from three independent experiments are shown.

Therefore, we examined whether drug-induced morphological alterations of schistosomula (Fig. 2) result in exposure of SmCD59a or SmKK7. Drug-treated schistosomula were incubated with anti-SmCD59a or anti-SmKK7 antiserum and analyzed by live imaging immunofluorescence microscopy. According to our observations and earlier reports [25,26] all three drugs were applied at 10 μM. The incubation time was 48 h for PZQ and ART and 6 days for OXA, as for the latter the anti-schistosomula effect can be delayed [27,28]. The experiments on SmCD59a were performed with 7- and 14-day schistosomula as we showed that these have the highest SmCD59a expression (Fig. 4). As shown in Fig. 8, OXA was most effective and triggered a bright fluorescence in about 80% of the 7-day as well as 14-day schistosomula. PZQ and ART led to exposure of SmCD59a in approximately 14% (PZQ) and 19% (ART) of the 14-day schistosomula. In 7-day schistosomula, PZQ and ART induced SmCD59a antigen exposure in about 5% of the parasites. To evaluate drug-induced exposure of SmKK7, 2-day schistosomula were included as SmKK7 expression was highest in these (Fig. 4). Binding of anti-SmKK7 antiserum was not detected in 7-day and 14-day schistosomula treated with PZQ, ART or OXA (S2 Fig.). Only PZQ and OXA were applied to 2-day schistosomula, because treatment with ART did not lead to morphological effects on the very young schistosomula (Fig. 2), which have not yet visible hemozoin in the guts (Fig. 1). Application of PZQ led to exposure of SmKK7 in less than 5% of the schistosomula (Fig. 9). Again, OXA was most effective and triggered fluorescence in nearly 80% of the schistosomula (Fig. 9). No signal was detected in untreated, undamaged schistosomula incubated with 0.1% DMSO as a negative control (Fig. 9). To assess antigen unmasking at lower drug doses, we analyzed the binding of anti-SmCD59a antiserum to schistosomula treated with 10, 1 and 0.1 μM OXA. As shown in Fig. 10, treatment with 1 μM OXA resulted in fluorescence signals in 40% of the schistosomula, and 0.1 μM OXA triggered a signal in 6% of the schistosomula. The extent of the observed effects varied depending on the vitality of the *in vitro*-cultured schistosomula (due to variations in culture conditions, i.e. in blood cell donors or FCS and medium batches). However, 1 μM of OXA turned out to be sufficient to expose antigens to antibody recognition. Interestingly, some schistosomula that survived 1 μM OXA treatment nevertheless showed antibody binding. This suggests that also larvae which were not instantly subject to fatal damage may have undergone alterations to structures near their surface allowing antibody attack (S9 Video). Taken together, the three drugs under study unmasked both antigens on *in vitr*o-cultured schistosomula, but to a differing extent with OXA being the most effective drug.

## Discussion

Most studies on *S*. *mansoni* focused on adult schistosomes or freshly transformed cercariae. Drug effects against schistosomula and antigen accessibility on the larval surface are still poorly understood. Here we undertook an in-depth analysis of the effects of the anthelmintic drugs PZQ, ART and OXA against these larvae at different time points of their development. Antibody accessibility of two exemplary antigens on viable larvae was analyzed to test their susceptibility to antibody-mediated attack. These studies were performed using *in vitro*-grown schistosomula which were generated and cultured on the basis of the protocol from Basch [12]. This *in vitro* culture system enables analysis of larvae at any desired time-point during their development. In addition, it imitates the host environment by providing red and white blood cells and soluble blood components to the parasites as a source of food and cytokines. This approach allows propagation of freshly transformed larvae up to adult schistosomes including pairing and deposition of immature eggs. Its greatest advantage is the use of human blood cells, mimicking a human host. Animal models, especially mouse models, are known for modifying drug effects via species-specific serum proteins [29].

Testing of three anthelmintics in this study demonstrated that PZQ applied at a concentration of 1 μM inhibited the development of larvae. Application of 10 μM PZQ led to dramatic morphological changes of all schistosomula. Juvenile and adult worms undergo a calcium-dependent contraction and paralysis following PZQ treatment [30]. However, unlike adults, juveniles survive 1 μM PZQ *in vitro* [31] (and own findings). Juveniles are thought to undergo similar calcium-binding and uptake mechanisms but experience distinct follow-up reactions that allow survival of immature worms only [32].

OXA is a pro-drug that is enzymatically converted into its active form by a sulfotransferase of schistosomes. Recently, Valentim et al. demonstrated loss-of-function mutations in the sulfotransferase of resistant parasites but not of OXA sensitive parasites [6]. The active form of OXA is supposed to act on the DNA level [3]. *In vivo* studies have shown that the effect of OXA occurs with a delay of 6 to 8 days [28], which is consistent with our findings *in vitro*.

ART, commercially used as a drug against malaria, inhibits the heme detoxification, which results in the generation of cytotoxic radical species and subsequent poisoning of the parasite. This mechanism of action is reflected by diverging ART effects on 2-day vs. 7- and 14-day schistosomula. Even at a concentration of 10 μM, ART is not lethal for 2-day schistosomula, which do not feed on blood yet: ART just inhibits their further development. In contrast, 7- and 14-day schistosomula, already having developed guts and feeding on erythrocytes, are severely damaged—supposedly due to intoxication by free heme.

Although schistosomicidal drugs do not prevent reinfection, it has been noted that multiple rounds of PZQ treatment are associated with growing levels of schistosome-specific antibodies and emergence of partial resistance against reinfection in humans [33–36]. The most probable explanation is that drug-mediated damage of schistosomes may uncover hidden surface antigens making them accessible to immune effector cells. Attempts in murine models to further substantiate this notion delivered inconsistent results. For example, PZQ treatment induced exposure of schistosome surface antigens [37–40], but was not sufficient to induce reproducible immunity [41,42]. For OXA treatment, results are also conflicting [28,43,44]. In a study using ART, induction of a protective immunity against *S*. *mansoni* was observed [45].

In the present study, PZQ, ART and OXA were investigated for their capacity to induce exposure of SmCD59a and SmKK7 on *in vitro*-cultured schistosomula. Of the three drugs, OXA induced the most pronounced effects with respect to parasite morphology and viability as well as antibody binding. Thus, incubation of schistosomula with 10 μM OXA for 6 days resulted in anti-SmCD59a antibody binding in about 80% of the parasites. PZQ and ART were less effective in exposing SmCD59a and SmKK7 but induced a prompt effect with the maximal drug-specific damage reached after incubation for 48 h.

The two antigens subject to this study seem to differ with respect to the time-course of protein expression: SmCD59a was expressed stronger in 7-day and 14-day schistosomula, whereas SmKK7 presented strongest in 2-day schistosomula. Immunofluorescence microscopy detected SmCD59a in formalin-fixed and permeabilized worms near the larval surface: SmCD59a presented diffusely on the schistosomula surface, while SmKK7 was condensed in spots and arranged as an internal network. The transcription of the SmCD59a gene was reported to be up-regulated during the transformation from cercariae to schistosomula [46]. That could explain that protein expression is still rather low in 2-day schistosomula but increases with further development. SmKK7 was found in cercariae and instead of being diffusely expressed, it has already been described to shape an internal network [15]. This arrangement might be explained by the assumption that SmKK7 is part of the peripheral nerval system [15,47]. Anti-SmCD59a and anti-SmKK7 did not bind to the surface of living intact 2-, 7- and 14-day *in vitro*-cultured schistosomula, whereas rat infection serum (RIS) used as a positive control did bind. Rats are able to overcome schistosome infection [48–51]. The underlying mechanisms have been described to involve complement activation and humoral immune response [49,50,52–54]. Rat infection serum proved to be positive for antibodies against several tegumental epitopes of living schistosomes [1,55]. In order to enable effective immune response, it is important to increase the exposure of antigens relevant for a humoral attack. As outlined above, clinically used anthelmintic drugs offer this possibility. Combining vaccination and drug therapy could speed up the process of acquiring resistance against reinfection.

In summary, we used *in vitro*-cultured schistosomula to investigate the drug-induced exposure of SmCD59a and SmKK7. Both antigens are largely hidden in untreated worms. We observed increased accessibility of these antigens after pharmacological treatment, which suggests that drug exposure renders schistosomes susceptible to immunogenic antibody attack.

## Supporting Information

S1 Video Effect of 0.1% DMSO on 14-day-schistosomula. *In vitro*-cultured schistosomula were treated with 0.1% DMSO in medium (solvent of the drugs) as negative control. DMSO was added at day 14 and schistosomula were analyzed at day 21.
(MP4)

S2 Video Schistosomicidal effect of PZQ on 14-day-schistosomula. *In vitro*-cultured schistosomula were treated with 10 μM PZQ. Drug was added at day 14 and schistosomula were analyzed at day 21 (late treatment).
(MP4)

S3 Video Schistosomicidal effect of ART on 14-day-schistosomula. *In vitro*-cultured schistosomula were treated with 10 μM ART. Drug was added at day 14 and schistosomula were analyzed at day 21 (late treatment).
(MP4)

S4 Video Schistosomicidal effect of OXA on 14-day-schistosomula. *In vitro*-cultured schistosomula were treated with 10 μM OXA. Drug was added at day 14 and schistosomula were analyzed at day 21 (late treatment).
(MP4)

S5 Video Binding of rat anti-SmCD59a antiserum to fixed and permeabilized schistosomula. Formalin-fixed and permeabilized schistosomula were analyzed at day 14 for SmCD59a localization. Video shows z-stack screening through schistosomula as assessed by confocal microsocopy.
(MP4)

S6 Video Binding of rat anti-SmKK7 antiserum to fixed and permeabilized schistosomula. Formalin-fixed and permeabilized schistosomula were analyzed at day 7 for SmKK7 localization. Video shows z-stack screening through schistosomula as assessed by confocal microsocopy.
(MP4)

S7 Video Binding of uninfected rat serum to fixed and permeabilized schistosomula. Formalin-fixed and permeabilized schistosomula were analyzed at day 7 with serum samples from uninfected rats (NRS) as negative control. Antibody binding was detected with a fluorophore-labeled anti-rat IgG antibody. Video shows z-stack screening through schistosomula as assessed by confocal microsocopy.
(MP4)

S8 Video Binding of infected rat serum to fixed and permeabilized schistosomula. Formalin-fixed and permeabilized schistosomula were analyzed at day 7 with serum samples from infected rats (RIS) as positive control. Antibody binding was detected with a fluorophore-labeled anti-rat IgG antibody. Video shows z-stack screening through schistosomula as assessed by confocal microsocopy.
(MP4)

S9 Video Binding of rat anti-SmCD59a antiserum to OXA treated schistosomula. 14-day schistosomula were treated with 1 μM OXA. 6 days after drug application schistosomula were incubated with anti-SmCD59a antiserum. Antibody binding was detected with a fluorophore-labeled anti-rat IgG.
(MP4)

S1 Fig Immunofluorescence reveals surface binding of damaged schistosomula by anti-SmCD59a antiserum. Living 14-day schistosomula were incubated with rat anti-SmCD59a antiserum. Surface binding of antibody was detected using fluorophore-labeled anti-rat IgG (IF). Binding to a damaged schistosomulum is highlighted by an arrow. Corresponding bright field image shows the developing schistosomula (BF). Scale bars = 200 μm.
(TIF)

S2 Fig Binding of anti-SmKK7 antiserum was not detected in 7-day and 14-day schistosomula treated with PZQ, ART or OXA. Drugs were added either at day 7 (A) or day 14 (B) and analyzed 2 days after administration of PZQ and ART and 6 days after OXA application. For the analysis of antigen accessibility, schistosomula were incubated with anti-SmKK7 antiserum. Serum sample from uninfected rats (NRS) was used as negative control. Antibody binding was detected with a fluorophore-labeled anti-rat IgG (IF). Corresponding lower images show the bright field images (BF). Scale bars = 200 μm.
(TIF)
