# Supplementary figures and images for: Drug-Induced Exposure of Schistosoma mansoni Antigens SmCD59a and SmKK7
Source: PLoS Negl Trop Dis. 2015 Mar 16;9(3):e0003593. doi: 10.1371/journal.pntd.0003593 (PMC4361651; doi:10.1371/journal.pntd.0003593)

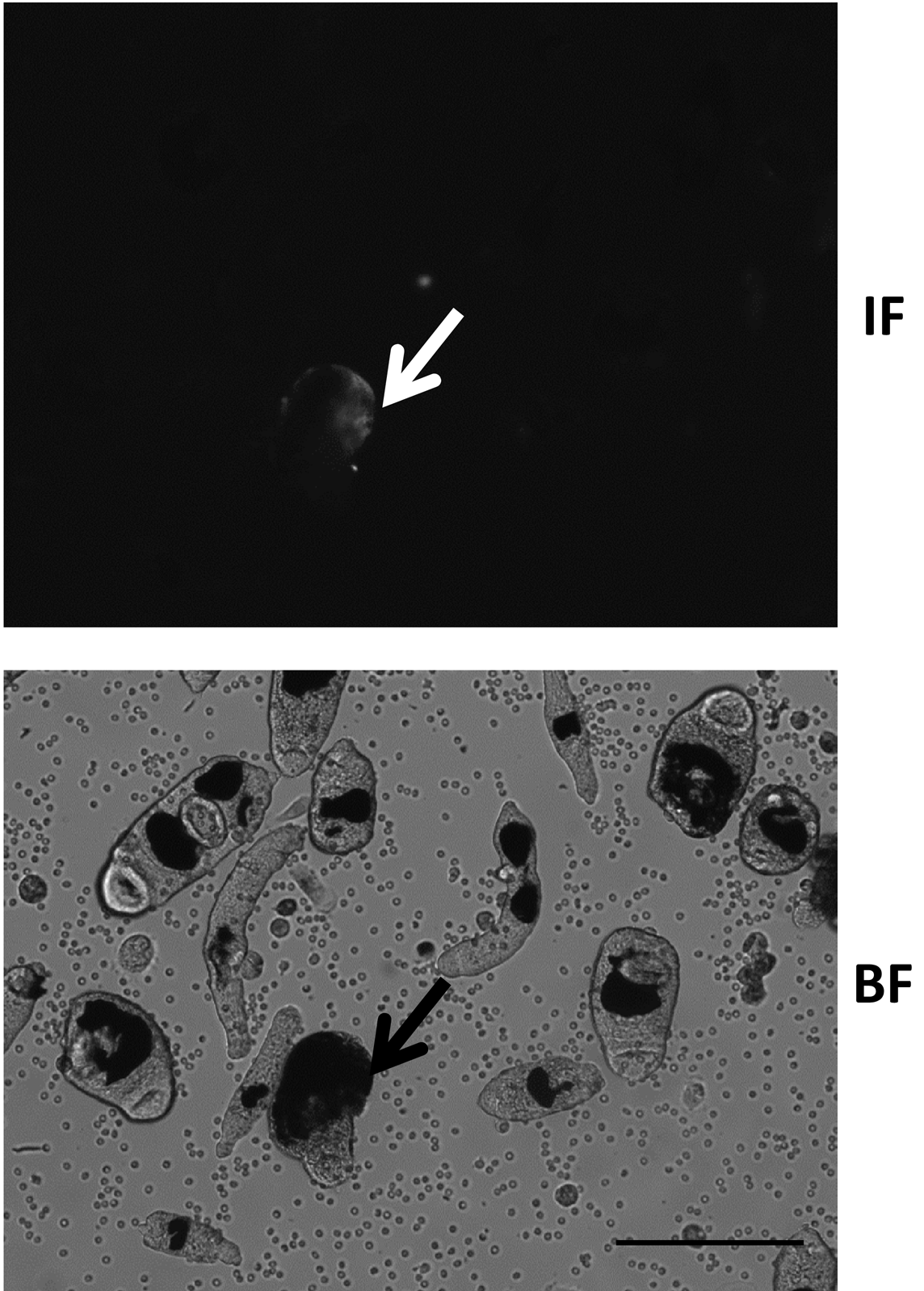

Supplement: S1 Fig — Living 14-day schistosomula were incubated with rat anti-SmCD59a antiserum. Surface binding of antibody was detected using fluorophore-labeled anti-rat IgG (IF). Binding to a damaged schistosomulum is highlighted by an arrow. Corresponding bright field image shows the developing schistosomula (BF). Scale bars = 200 μm. (TIF) [file pntd.0003593.s010.tif]

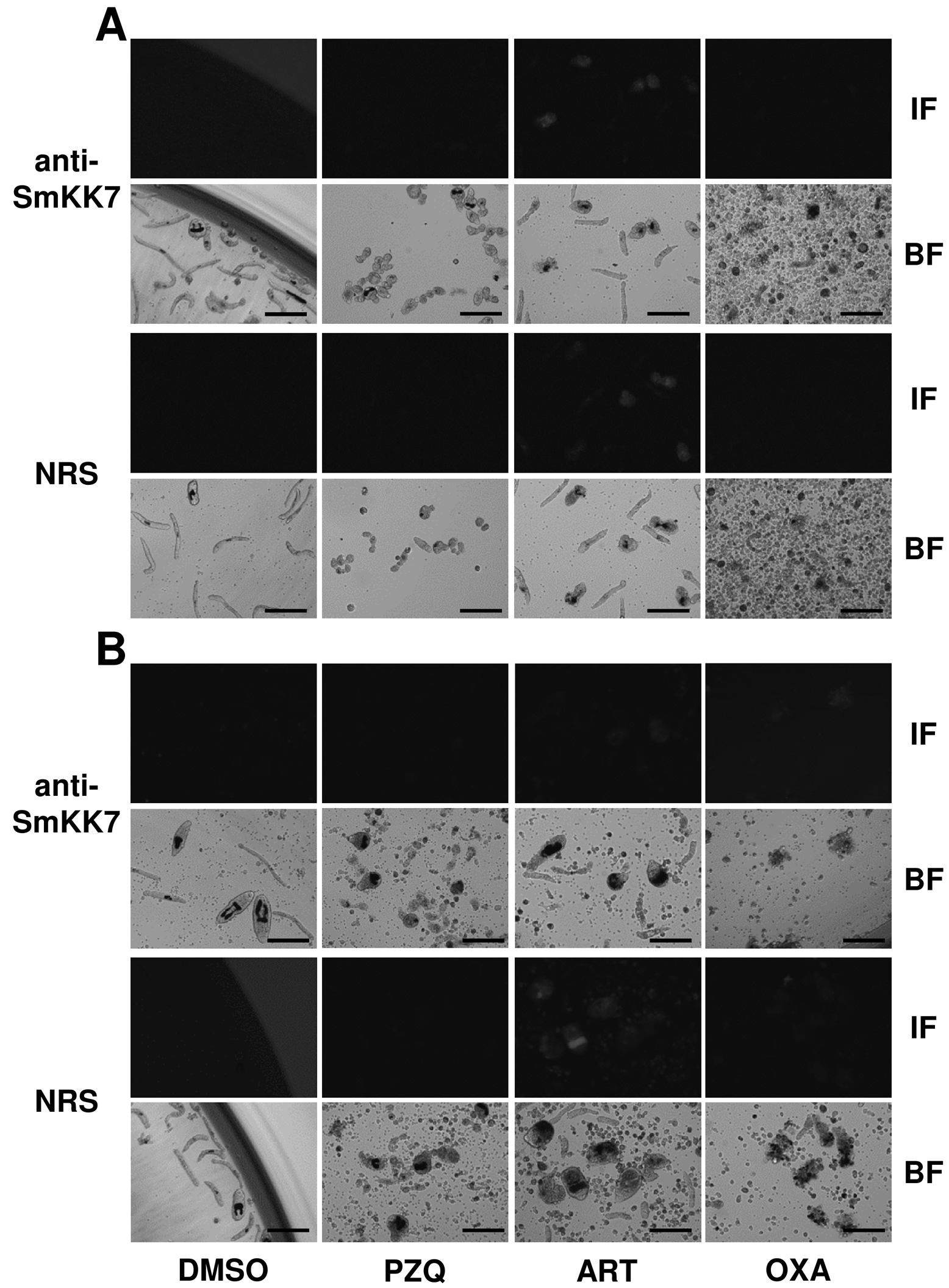

Supplement: S2 Fig — Drugs were added either at day 7 (A) or day 14 (B) and analyzed 2 days after administration of PZQ and ART and 6 days after OXA application. For the analysis of antigen accessibility, schistosomula were incubated with anti-SmKK7 antiserum. Serum sample from uninfected rats (NRS) was used as negative control. Antibody binding was detected with a fluorophore-labeled anti-rat IgG (IF). Corresponding lower images show the bright field images (BF). Scale bars = 200 μm. (TIF) [file pntd.0003593.s011.tif]
